# Unique characteristics of G719X and S768I compound double mutations of epidermal growth factor receptor (*EGFR*) gene in lung cancer of coal-producing areas of East Yunnan in Southwestern China

**DOI:** 10.1186/s41021-022-00248-z

**Published:** 2022-05-23

**Authors:** Jun-Ling Wang, Yu-Dong Fu, Yan-Hong Gao, Xiu-Ping Li, Qian Xiong, Rui Li, Bo Hou, Ruo-Shan Huang, Jun-Feng Wang, Jian-Kun Zhang, Jia-Ling Lv, Chao Zhang, Hong-Wei Li

**Affiliations:** 1 Biological Laboratory, First People’s Hospital of Qujing, Qujing, 655000 China; 2 Department of Thoracic Surgery, First People’s Hospital of Qujing, Qujing, 655000 China; 3 Department of Traditional Chinese Medicine, First People’s Hospital of Qujing, Qujing, 655000 China; 4 Department of Medical Administration, First People’s Hospital of Qujing, Qujing, 655000 China; 5 Department of Pathology, First People’s Hospital of Qujing, Qujing, 655000 China; 6 Department of Oncology, First People’s Hospital of Qujing, Qujing, 655000 China

**Keywords:** Lung cancer, *EGFR* gene mutation, Coal-producing areas, G719X and S768I compound double mutation

## Abstract

**Background:**

The principal objective of this project was to investigate the *Epidermal Growth Factor Receptor* (*EGFR*) gene mutation characteristics of lung cancer patients, which can provide a molecular basis for explaining the clinicopathological features, epidemiology and use of targeted therapy in lung cancer patients in the coal-producing areas of East Yunnan.

**Methodology:**

We collected 864 pathologically confirmed lung cancer patients’ specimens in First People’s Hospital of Qujing City of Yunnan Province from September 2016 to September 2021. We thereafter employed Next Generation Sequencing (NGS) technology to detect all exons present in the *EGFR* gene.

**Results:**

The overall mutation frequency of the *EGFR* gene was 47.22%. The frequency of *EGFR* gene mutations in the tissue, plasma, and cytology samples were found to be 53.40%, 23.33%, and 62.50%, respectively. Univariate analysis indicated that the coal-producing areas and Fuyuan county origin were significantly associated with relatively low *EGFR* gene mutation frequency. Female, non-smoking history, adenocarcinoma, non-brain metastasis, and tissue specimens were found to be related to high *EGFR* gene mutation frequency. Multivariate logistic regression analysis suggested the lung cancer patients in the central area of Qujing City, stage Ia, non-coal-producing areas, non-Fuyuan origin, and non-Xuanwei origin were more likely to develop *EGFR* gene mutations. The most common mutations were L858R point mutation (33.09%) and exon 19 deletion (19-del) (21.32%). Interestingly, the mutation frequency of G719X (*p* = 0.001) and G719X + S768I (*p* = 0.000) in the coal-producing areas were noted to be more significant than those in non-coal-producing regions.

**Conclusion:**

This findings of this study might be important in establishing the correlation between routine using NGS for *EGFR* gene mutation diagnosis and clinical practice in the lung cancer patients.

**Supplementary Information:**

The online version contains supplementary material available at 10.1186/s41021-022-00248-z.

## Introduction

Lung cancer is one of the major factors for cancer death worldwide, including in China. Approximately 2.09 million new lung cancer cases are diagnosed every year worldwide, leading to over 1,761,000 deaths [1]. In 2015, 733,300 new lung cancer cases and 610,200 lung cancer deaths were reported in China [2]. Non-small-cell lung cancer (NSCLC) accounts for about 85% of all the lung cancers, and lung adenocarcinoma (LUAD) is the most common subtype [3]. It has been found that compared with other parts of China, lung cancer in Xuanwei county of Qujing city of Yunnan province was most severe in the countryside, where women almost had no smoking history. Still, the overall incidence rate of lung cancer in Xuanwei county is 4–5 times higher than in other regions, and the mortality rate is as high as 91.3 per 100,000 persons [4, 5]. The main features of the lung cancer in Xuanwei County included higher incidence of non-smokers females, younger age at diagnosis, rapid tumor progression, presence of more lung lesions, poor prognosis, and familial aggregation [6]. These areas with substantially high incidence rates of the lung cancer are primarily located in eastern and northern Yunnan, western Guizhou, where the Late Permian coal accumulated areas and abounds with Bituminous Coal, which can emit the various polycyclic aromatic hydrocarbons (PAHs), silica, heavy metal elements, inhalable particulate matter, and other carcinogens after burning [7]. A retrospective study has previously demonstrated that the lifelong smoky coal consumption increased mortality by 99 fold in women compared to the smokeless coal use [8]. Generally, the lung cancer patients in the coal-producing areas might present a different subgroup globally, which has prompted researchers to detect the various tumors mutations through next-generation sequencing (NGS) to discover specific gene mutation sites to facilitate the application of suitable targeted therapy. In addition, these extraordinary mutation sites of the driver gene may aid to explain the novel concept of lung cancer epidemiological and clinicopathological characteristics in the coal-producing areas.

Epidermal growth factor receptor (*EGFR*) is one of the most common driver genes involved in the lung cancer mutation, which can regulate activation of both phosphatidylinositol 3-kinase/protein kinase B/mammalian target of rapamycin (PI3K/AKT/mTOR), and mitogen-activated protein kinase (MAPK) signaling pathways. The remarkable advent of gefitinib in 2000, EGFR tyrosine kinase inhibitor (EGFR-TKI) targeted therapy as a representative precision medicine has facilitated spectacular alterations to the lung cancer study philosophy and treatment model. It also enabled the various targeted agents, which can effectively inhibit EGFR to undergo multiple generations of clinical development. Currently, China, South Korea, and the U.S. Food and Drug Administration have approved EGFR-TKIs of 4 generations of different 11 kinds. The best objective response rate (ORR), disease control rate (DCR), and progression-free survival (PFS) in response to these targeted agents was found to be 87%, 93.6%, and 19.4 months, respectively [9].

The frequency of *EGFR* gene mutation was significantly different among the lung cancer patients of the various races or ethnic groups. A number of the previous studies have documented that the mutation rate of the *EGFR* gene in North American and European populations was 10%-15%, whereas the mutation rate in various East Asians, including Chinese, Korean, and Japanese, was 20%-76% [10]. A recent study has shown that the overall *EGFR* gene mutation frequency in Yunnan province was about 39.47–46.2% [11, 12]. In addition, rare *EGFR* gene G719X single mutation and G719X + S768I co-mutation were the primary *EGFR* gene mutations identified in the Xuanwei NSCLC cohort. Moreover, the co-mutation rate of *EGFR *exon 18 and 20 in the Xuanwei NSCLC cohort was also significantly higher than that of other regions [8, 12, 13]. However, the above findings were limited to only one coal-producing area in Xuanwei County. The detailed characteristics of *EGFR* gene mutations in the lung cancer patients in other coal-producing areas in Eastern Yunnan are still unclear. In this study, we have examined the *EGFR* gene mutation frequency of 864 different lung cancer patients in the surrounding area of the Qujing City of Yunnan Province by using the method of next-generation sequencing. We have further compared the frequency of *EGFR *gene mutation between the lung cancer cohort in the coal-producing areas and those of the non-coal-producing areas. Finally, correlation studies were conducted to establish the potential link between *EGFR* gene mutation and demographic and clinicopathological characteristics.

## Material and methods

### Patients and regions distribution

We have collected 864 tumor samples from stages Ia-IV lung cancer patients treated in the First People’s Hospital of Qujing City of Yunnan Province between September 2016 to September 2021. Eligibility criteria used were as follows: (1) adults (> 18 years) who were dwelling in Eastern Yunnan province, (2) pathological confirmed lung cancer patients. The Ethical Committee of First People’s Hospital of Qujing City approved this study protocol (approval number 2016–023-01). All the participants were required to sign an institutional review board-approved informed consent. 864 lung cancer patients from east Yunnan, the central nine counties, were enrolled in this study. We divided the eastern Yunnan region into the coal-producing and non-coal-producing areas based on the basic location table of Qujing coal mines provided by the Qujing Coal Industry Bureau (Supplementary Table 1).

### Samples and DNA extraction

We tested 864 tumor samples by using the next-generation sequencing approach for discovering lung cancer *EGFR* gene mutations, including formaldehyde fixed paraffin embedded (FFPE) tumor tissues from the surgical resections (614 cases), plasma (180 cases), FFPE tumor tissue from biopsies (37 cases), fresh tumor tissue from surgical resections (13 cases), fresh tumor tissue from the biopsies (12 cases), and malignant pleural effusion (8 cases). The tissue specimens were obtained from the surgical resection specimens and biopsy specimens (bronchoscope biopsy, transbronchial lung biopsy, percutaneous needle biopsy, pleural biopsy, and metastasis biopsy). The cytological examination was primarily obtained from the pleural effusion. First, we performed Hematoxylin and Eosin staining on the samples to detect the potential content of the tumor cells (more than 20% tumors) and then performed nucleic acid extraction on qualified samples. The blood was collected in 10 ml BD Vacutainer K2 EDTA tubes (BD Biosciences, New York, USA) and then centrifuged at 1500 rpm for 20 min at 4 °C within 2 h. Subsequently, the supernatant was harvested and centrifuged at 13,000 rpm for 10 min at 4 °C. Thereafter, the plasma was frozen for storage at − 80 °C or immediately used for the circulating tumor DNA extraction. According to the manufacturer’s instructions, we used the QIAGEN DNeasy Blood & Tissue Kit and QIAamp circulating nucleic acid kit (Qiagen, Frankfurt, Germany) to extract the genomic DNA from tissues, plasma, and cytology. After extraction, the DNA concentration was evaluated with the Qubit 3.0 Fluorometer (Life Technologies, California, USA), by utilizing the Qubit dsDNA BR Assay Kit (Invitrogen, California, USA).

### Library construction

First, more than 10 ng of genomic DNA was broken into an average fragment size of 300 base pairs by using the Covaris ultrasonic disruptor (E210, Covaris Inc., Massachusetts, USA). Thereafter, the sequencing libraries were prepared with the Accel-NGS® 2S Hyb DNA Library Kit (Swift Biosciences, California, USA), including end repair, base addition, and adaptor ligation steps. KAPA HiFi HotStart ReadyMix PCR Kit (Kapa Biosystems, Boston, USA) was adopted for the library enrichment. The PCR-amplified DNA-seq library quality was thereafter assessed using the Agilent DNA 1000 kit (Agilent Technologies, California, USA) on the Agilent 2100 BioAnalyzer (Agilent Technologies, California, USA) and quantified by using Qubit 3.0 Fluorometer (Life Technologies, California, USA).

### Hybridization capture and sequencing

After analyzing both the quality and quantity of the amplified library, more than 200 ng selected library pool were hybridized with a custom panel of xGen Lockdown Probes (IDT) for targeted gene *EGFR* (IDT DNA, USA), which was then allowed to incubate overnight at 65 °C for 16 h. Thereafter, the hybrid library was washed according to the manufacturer’s protocols of the NimbleGenSeqCap EZ Hybridization and Wash kit (NimblegenSeqCap EZ Human Exome Library v.2.0). Next, we conducted the post-capture library amplification. The hybrid library was generated using the relevant components of Illumina’s Nextera Rapid Capture Exome Kit and by following the manufacturer’s suggested protocol. The amplified hybrid library concentrations were assessed by using quantitative PCR using the KAPA Library Quantification Kit (Kapa Biosystems, Boston, USA). All the qualified libraries were sequenced on the NovaSeq 6000 Sequencing System (Illumina, California, USA). The quality assessment standards of the sequencing were that: (1) mean sequencing depth coverage across the different tissue and plasma samples were 500 × and 1500 × , respectively, (2) the threshold for the quality filtering was adjusted to > Q30 across 90%, (3) the mapping efficiency of the sequence data to a reference genome more than 95%, and (4) the libraries with hybrid capture efficiency more than 40%.

### Sequence data processing

After sequencing, the raw fastq files were quality-filtered by using Trimmomatic and merged by Fast Length Adjustment of Short reads (FLASH, http://www.cbcb.umd.edu/software/flash). The various sequence reads were mapped to the reference genome (hg19: GRCh37: Feb2009) by using the Burrows-Wheeler Aligner (BWA) (version 0.7.1). The variant detection was performed using the HaplotypeCaller in the Genome Analysis Took Kit (GATK) package (3.8–0), and the results were annotated by ANNOVAR (Qiagen, Frankfurt, Germany). The somatic mutations were selected by searching for variants with an alternate allele fraction of at least 0.2% and at least 5 supporting reads. The common single nucleotide polymorphisms (SNPs) were screened with dbSNP (v137) and the 1000 Genomes database. The variants were filtered for common SNPs from the most current dbSNP database and 1000 Genomes Project. We adopted an ONCOCNV package to detect the copy number aberrations (CNAs) in the targeted deep sequencing data (Pairs, France, https://oncocnv.curie.fr).

### Statistical analysis

Pearson chi-square tests were used to analyze the relationship between the clinical demographic factors and *EGFR* gene mutation (age, gender, smoking history, histological type, region distribution, nationality, specimen type, tumor site, coal-producing area, Fuyuan origin, and Xuanwei origin). Similarly, multivariable binary logistic regression models were used for the binary outcomes. All the statistical analyses were performed using SPSS Statistics software (version 22.0, SPSS Inc). The difference was considered as statistically significant when *p* < 0.05. *P*-values were set at 0.01 (*p* < 0.01) for highly significant difference, and 0.001 (*p* < 0.001) for extremely significant difference.

## Results

### Clinical characteristics

**Fig. 1:**
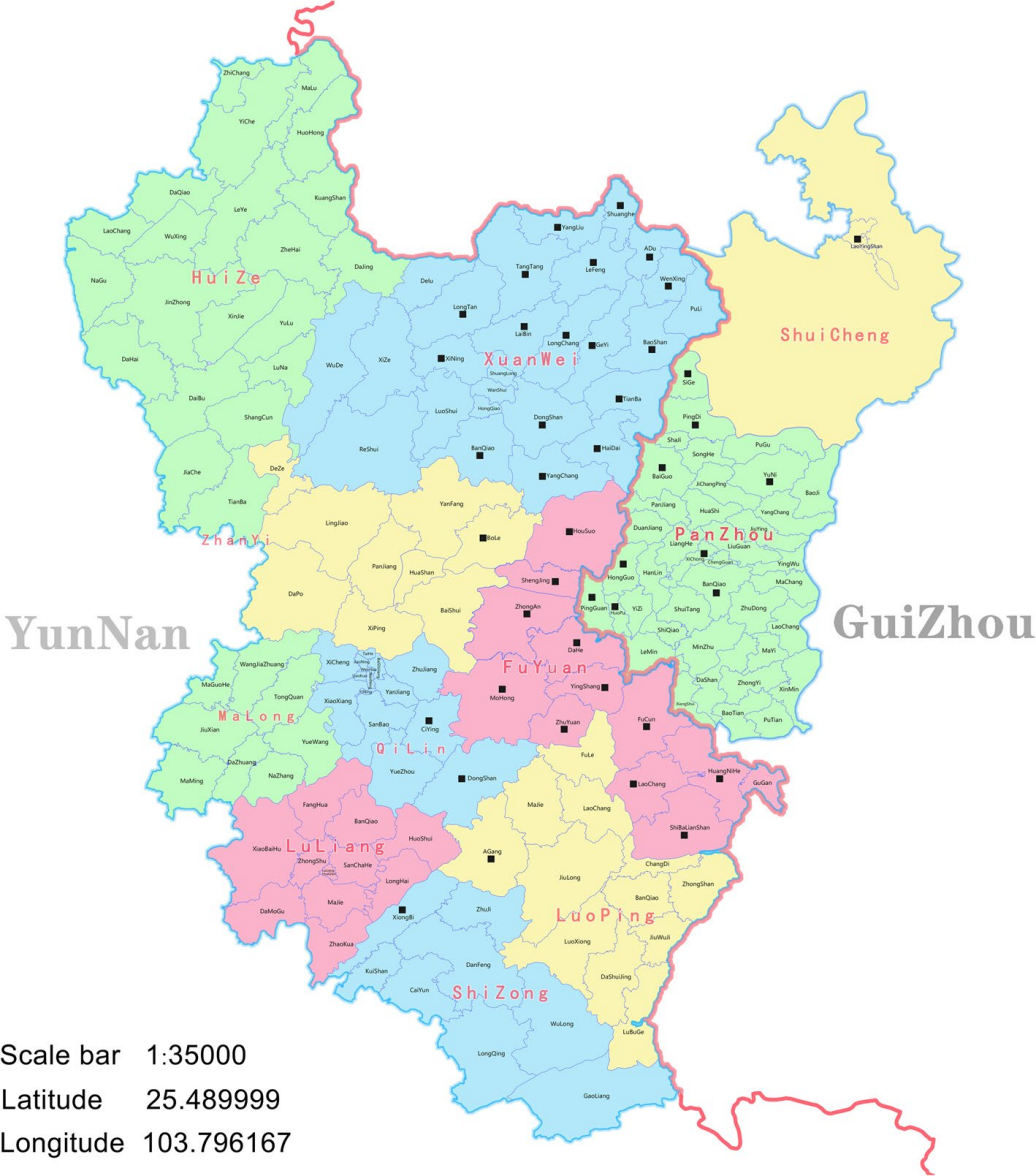
A map of 9 counties in Qujing City has been shown to the township. The solid black square (■) indicates the different towns in the coal-producing areas. The Yunnan province map is on the left, and the Guizhou province map is on the right

864 pathologically confirmed lung cancer patients were enrolled in our research. Table 1 summarizes the baseline characteristics of all the patients involved in this study. Among these patients, 458 cases (53.01%) were females, and 406 cases (46.99%) were males. The mean age of females was 56 ± 9.55 years, ranging from 26 to 91 years. The mean age of males was 57 ± 10.34 years, ranging from 19 to 89 years. In addition, 203 patients (23.50%) had a history of smoking, and 661 patients (76.50%) had never smoked previously. One hundred fifty-seven patients (18.17%) had a family history of the malignant tumors. The most prevalent histological type was that of the lung adenocarcinoma (757 cases, 87.62%), followed by the squamous cell carcinoma (29 cases, 3.36%), adenosquamous carcinoma (3 cases, 0.35%), and the large cell carcinoma (5 cases, 0.58%), whereas 70 cases (8.10%) of NSCLC were undefined. Moreover, 326 patients (37.73%) had tumors on the left side of the lung, 514 patients (59.49%) developed tumors on the right side of the lung, and 24 patients (2.78%) had tumors on the bilateral lungs. It was observed that among the nine different areas in Eastern Yunnan (Fig. 1), 230 patients (26.62%) were native to the central region (Qilin), 254 patients (29.40%) were native to the east (Fuyuan), and 207 patients (23.96%) were native to the northeast (Xuanwei (190 cases, 21.99%), Panzhou (17 cases, 1.97%)), 11 patients (1.27%) were native to the southeast (Luoping), 14 patients (1.62%) were native to the south (Shizong), 26 patients (3.01%) were native to the west (Malong), only eight patients (0.93%) were native to the northwest (Huize), and 16 patients (1.85%) were native to the southwest (Luliang), 65 patients (7.52%) were native to the north (Zhanyi), and the other 33 patients (3.82%) were native to non-Eastern Yunnan. Of all 864 patients, 522 patients (60.42%) belonged to the coal-producing belts in Eastern Yunnan, of which 254 patients (29.40%) grew out of Fuyuan County, and the remaining 190 patients were from Xuanwei county, where is a renowned coal yield county in the east of Yunnan with the highest incidence and mortality of lung cancer in the rural areas all over the world. The Han ethnic group was the most common among the enrolled subjects (859 people, 99.42%), and the minority Yi nationality consisted of merely five people (0.58%). According to the TNM staging system, 656 patients (75.93%) were diagnosed in stage Ia-IIIa, and 208 patients (24.07%) belonged to the stage IIIb-IV. There were 180 patients (20.83%) with brain metastases and 684 (79.17%) patients without brain metastases. In our study, the vast majority of lung cancer patients underwent surgery at an early stage and hence we could obtain their tissue specimens, while for patients who were not subjected to surgery only cytological specimens and needle biopsy specimens were used. The most commonly used specimen in this study was the tissue specimens (676 cases, 78.24%), followed by the plasma specimens (180 cases, 20.83%), and the least was the cell based specimens (8 cases, 0.93%). Five hundred and twenty-two (60.42%) lung cancer patients belonged to the coal-producing regions, and the remaining people came from the non-coal-producing regions (342 cases, 39.58%).

**Table 1:** Frequency of *EGFR* mutation as reported by clinical features in the general patients

	Sum total	Positive		Negative			*P*
		N	%		N	%	
**Age**							0.070
< 65	672	327	48.66%		345	51.34%	
65–75	166	74	44.58%		92	55.42%	
> 75	26	7	26.92%		19	73.08%	
**Gender**							**0.000**
Male	406	150	36.95%		256	63.05%	
Female	458	258	56.33%		200	43.67%	
**Smoking history**							**0.000**
Yes	203	64	31.53%		139	68.47%	
No	661	344	52.04%		317	47.96%	
**Family history of malignant tumors**							0.206
Yes	157	69	43.95%		88	56.05%	
No	707	339	47.95%		368	52.05%	
**Histological and pathological type**							**0.000**
AD	757	376	49.67%		381	50.33%	
SCC	29	5	17.24%		24	82.76%	
ADSC	3	3	100.00%		0	0.00%	
LCC	5	1	20.00%		4	80.00%	
NSCLC	70	23	32.86%		47	67.14%	
**Tumor site**							0.356
Left	326	158	48.47%		168	51.53%	
Right	514	242	47.08%		272	52.92%	
Bilateral	24	8	33.33%		16	66.67%	
**Regional distribution**							0.092
Central	230	125	54.35%		105	45.65%	
East	254	103	40.55%		151	59.45%	
Northeast	207	91	43.96%		116	56.04%	
Southeast	11	7	63.64%		4	36.36%	
South	14	6	42.86%		8	57.14%	
West	26	11	42.31%		15	57.69%	
Northwest	8	5	62.50%		3	37.50%	
Southwest	16	7	43.75%		9	56.25%	
North	65	37	56.92%		28	43.08%	
Other	34	16	48.48%		17	51.52%	
**Ethnic group**							0.552
Han	859	406	47.26%		453	52.74%	
Yi	5	2	40.00%		3	60.00%	
**TNM staging**							0.198
Ia	206	93	45.15%		113	54.85%	
Ib	177	85	48.02%		92	51.98%	
IIa	139	65	46.76%		74	53.24%	
IIb	97	54	55.67%		43	44.33%	
IIIa	37	21	56.76%		16	43.24%	
IIIb	41	13	31.71%		28	68.29%	
IV	167	77	46.11%		90	53.89%	
**Brain metastasis**							**0.000**
Yes	180	60	33.33%		120	66.67%	
No	684	348	50.88%		336	49.12%	
**Specimen type**							**0.000**
Tissue	676	361	53.40%		315	46.60%	
Plasma	180	42	23.33%		138	76.67%	
Cytology	8	5	62.50%		3	37.50%	
**Coal producing area**							**0.043**
Yes	522	232	44.44%		290	55.56%	
No	342	176	51.46%		166	48.54%	
**Fuyuan County origin**							**0.007**
Yes	254	103	40.55%		151	59.45%	
No	610	305	50.00%		305	50.00%	
**Xuanwei County origin**							0.088
Yes	190	81	42.63%		109	57.37%	
No	674	327	48.52%		347	51.48%	
**Total**	864	408	47.22%		456	52.78%	

*AD* adenocarcinoma, *SCC* squamous-cell carcinoma, *ADSC* adenosquamous carcinoma, *LCC* large cell carcinoma, *NSCLC* non-small-cell lung cancer

### The incidence of EGFR gene mutation and its correlation with the various demographic and clinical factors

The incidence of *EGFR* gene mutation and its correlation with clinicopathological parameters in the lung cancer patients in Eastern Yunnan was found to be similar to the overall situation of Yunnan Province. It was observed that 408 cases of *EGFR* gene mutation (47.2%) were detected in 864 patients with the lung cancer. The *EGFR* gene mutation rate varied significantly with gender, smoking status, pathological type, brain metastasis, specimen type, irrespective of the location and whether the patient came from a coal-producing area or from Fuyuan county. Female (*p* < 0.001), non-smoker (*p* < 0.001), adenocarcinoma (*p* < 0.001), non-brain metastasis (*p* < 0.001), tissue specimens (*p* < 0.001), patient from the coal-producing area (*p* < 0.05) and non-Fuyuan country origin patients (*p* = 0.007) appeared to be associated with a higher *EGFR* gene mutation rate. However, no significant correlation was found in age (*p* = 0.070), family history of malignancy (*p* = 0.206), tumor site (*p* = 0.356), regional distribution (*p* = 0.092), nationality (*p* = 0.552), TNM staging (*p* = 0.198) and Xuanwei county origin patients (*p* = 0.088) (Table 1).

Multivariate logistic regression analysis illustrated that the regional distribution (*p* < 0.001), TNM staging (*p* < 0.001), whether from a coal-producing area (*p* = 0.018), Fuyuan county origin (*p* < 0.001) or Xuanwei county origin (*p* < 0.001) acted as independent influencing factors of *EGFR* gene mutation (Table 2).

**Table 2:** Multivariate logistic regression analysis of the correlation between *EGFR* gene mutation and clinicopathological characteristics of the lung cancer patients

Factor	*β*	Wald χ 2	OR	95% CI	*P *value
Regional distribution	-1.872	41.843	0.154	0.087–0.271	0.000
TNM staging	-1.786	49.691	0.168	0.102–0.276	0.000
Coal producing area	1.034	5.607	2.813	1.195–6.619	0.018
Fuyuan County origin	-15.459	53.358	0.000	0.000–0.000	0.000
Xuanwei County origin	-11.939	43.654	0.000	0.000–0.000	0.000

### Incidence of EGFR gene mutation in the coal-producing areas and the non-coal-producing areas

Subgroup analysis indicated that the mutation rate of the *EGFR* gene of the lung cancer patients in non-coal-producing areas was 51.46% (176/342) higher than that of the coal-producing regions (44.44%, 232/522). Among the lung cancer patients in the coal-producing regions, females (*p* < 0.001), non-smokers (*p* < 0.001), and non-Fuyuan county origin (*p* < 0.05) appeared to be associated with significantly higher *EGFR* gene mutation frequency. However, among the various patients with lung cancer in the non-coal-producing regions, women (*p* < 0.001), non-smokers (*p* = 0.003), adenocarcinoma (*p* < 0.001), TNM stage I-IIIa (*p* = 0.015), and non-brain metastases (*p* < 0.001), nevertheless, appeared to be connected with a more unusual *EGFR* gene mutation frequency (Table 3).

**Table 3:** Comparison of *EGFR* gene mutation frequency in the non-coal-producing areas subgroups and the coal-producing areas

	Coal-producing areas						Non-coal-producing areas					
	Sum total	Positive		Negative		*p*	Sum total	Positive		Negative		*P*
		N	%	N	%			N	%	N	%	
**Age**						0.107						0.317
< 65	427	197	46.14	230	53.86		245	130	53.06	115	46.94	
65–75	84	33	39.29	51	60.71		82	41	50.00	41	50.00	
> 75	11	2	18.18	9	81.82		15	5	33.33	10	66.67	
**Gender**						**0.000**						**0.000**
Male	253	92	36.36	161	63.64		153	58	37.91	95	62.09	
Female	269	140	52.04	129	47.96		189	118	62.43	71	37.57	
**Smoking history**						**0.000**						**0.003**
Yes	134	39	29.10	95	70.90		69	25	36.23	44	63.77	
No	388	193	49.74	195	50.26		273	151	55.31	122	44.69	
**Family history of malignant tumors**						0.239						0.508
Yes	119	49	41.18	70	58.82		38	20	52.63	18	47.37	
No	403	183	45.41	220	54.59		304	156	51.32	148	48.68	
**Histological and pathological type**						0.076						**0.000**
AD	462	211	45.67	251	54.33		295	165	55.93	130	44.07	
Non-AD	60	21	35.00	39	65.00		47	11	23.40	36	76.60	
**Tumor site**						0.612						0.470
Left	198	92	46.46	106	53.54		128	66	51.56	62	48.44	
Right	307	134	43.65	173	56.35		207	108	52.17	99	47.83	
Bilateral	17	6	35.29	11	64.71		7	2	28.57	5	71.43	
**Regional distribution**						0.167						0.846
Central	65	39	60.00	26	40.00		165	86	52.12	79	47.88	
East	254	103	40.55	151	59.45		0	0	0.00	0	0.00	
Northeast	173	77	44.51	96	55.49		34	14	41.18	20	58.82	
Southeast	1	1	100.00	0	0.00		10	6	60.00	4	40.00	
South	6	2	33.33	4	66.67		8	4	50.00	4	50.00	
West	0	0	0.00	0	0.00		26	11	42.31	15	57.69	
Northwest	0	0	0.00	0	0.00		8	5	62.50	3	37.50	
Southwest	1	0	0.00	1	100.00		15	7	46.67	8	53.33	
North	8	4	50.00	4	50.00		57	33	57.89	24	42.11	
Other	14	6	42.86	8	57.14		19	10	52.63	9	47.37	
**Ethnic group**						0.400						0.515
Han	518	231	44.59	287	55.41		341	175	51.32	166	48.68	
Yi	4	1	25.00	3	75.00		1	1	100.00	0	0.00	
**TNM staging**						0.434						**0.015**
I-IIIa	381	168	44.09	213	55.91		275	150	54.55	125	45.45	
IIIb-IV	141	64	45.39	77	54.61		67	26	38.81	41	61.19	
**Brain metastasis**						0.110						**0.000**
Yes	46	16	34.78	30	65.22		55	8	14.55	47	85.45	
No	476	216	45.38	260	54.62		287	168	58.54	119	41.46	
**Xuanwei County origin**						0.331						0.182
Yes	157	67	42.68	90	57.32		33	14	42.42	19	57.58	
No	365	165	45.21	200	54.79		309	162	52.43	147	47.57	
**Fuyuan County origin**						**0.049**						NA
Yes	254	103	40.55	151	59.45		0	0	0.00	0	0.00	
No	268	129	48.13	139	51.87		342	176	51.46	166	48.54	
**Total**	522	232	44.44	290	55.56		342	176	51.46	166	48.54	

### Frequency of EGFR gene mutation in the tissue, plasma, and adenocarcinoma subgroups

Since the sample type may be an important confounding factor for *EGFR* gene testing, we separately investigated the incidence of *EGFR* gene mutation in the tissues and plasma. It was noted that *EGFR* gene mutation rate in the tissues was 53.40% (361/676), and the *EGFR* gene mutation rate in the plasma and pleural effusion cells was 25.00% (47/188). In the tissue subgroup, females (*p* < 0.001), non-smokers (*p* < 0.001), adenocarcinoma (*p* = 0.003), patients from the coal-producing areas, and non-Fuyuan county origin were found to be associated with higher *EGFR* gene mutation rates. In the plasma subgroup, we observed that only women (*p* = 0.014) and non-smokers (*p* = 0.011) were associated with a higher *EGFR* gene mutation rate (Table 4).

**Table 4:** Incidence of *EGFR* gene mutation in the tissue and plasma subgroups

	Tissue						Plasma and pleural effusion cells					
	Sum total	Positive		Negative		*p*	Sum total	Positive		Negative		*p*
		N	%	N	%			N	%	N	%	
**Age**						0.520						0.894
< 65	551	296	53.72	255	46.28		121	31	25.62	90	74.38	
65–75	114	61	53.51	53	46.49		52	13	25.00	39	75.00	
> 75	11	4	36.36	7	63.64		15	3	20.00	12	80.00	
**Gender**						**0.000**						**0.014**
Male	314	134	42.68	180	57.32		92	16	17.39	76	82.61	
Female	362	227	62.71	135	37.29		96	31	32.29	65	67.71	
**Smoking history**						**0.000**						**0.011**
Yes	159	59	37.11	100	62.89		44	5	11.36	39	88.64	
No	517	302	58.41	215	41.59		144	42	29.17	102	70.83	
**Family history of malignant tumors**						0.148						0.598
Yes	125	61	48.80	64	51.20		32	8	25.00	24	75.00	
No	551	300	54.45	251	45.55		156	39	25.00	117	75.00	
**Histological and pathological type**						**0.003**						0.459
AD	626	344	54.95	282	45.05		131	32	24.43	99	75.57	
Non-AD	50	17	34.00	33	66.00		57	15	26.32	42	73.68	
**Tumor site**						0.532						0.647
Left	252	137	54.37	115	45.63		74	21	28.38	53	71.62	
Right	411	219	53.28	192	46.72		103	23	22.33	80	77.67	
Bilateral	13	5	38.46	8	61.54		11	3	27.27	8	72.73	
**Regional distribution**						0.102						0.192
Central	190	110	57.89	80	42.11		40	15	37.50	25	62.50	
East	197	90	45.69	107	54.31		57	13	22.81	44	77.19	
Northeast	158	81	51.27	77	48.73		49	10	20.41	39	79.59	
Southeast	8	6	75.00	2	25.00		3	1	33.33	2	66.67	
South	11	5	45.45	6	54.55		3	1	33.33	2	66.67	
West	20	11	55.00	9	45.00		6	0	0.00	6	100.00	
Northwest	7	4	57.14	3	42.86		1	1	100.00	0	0.00	
Southwest	12	5	41.67	7	58.33		4	2	50.00	2	50.00	
North	53	36	67.92	17	32.08		12	1	8.33	11	91.67	
Other	20	13	65.00	7	35.00		13	3	23.08	10	76.92	
**Ethnic group**						0.635						0.750
Han	672	359	53.42	313	46.58		1	0	0.00	1	100.00	
Yi	4	2	50.00	2	50.00		187	47	25.13	140	74.87	
**TNM staging**						0.447						0.083
I-IIIa	549	292	53.19	257	46.81		114	33	28.95	81	71.05	
IIIb-IV	127	69	54.33	58	45.67		74	14	18.92	60	81.08	
**Brain metastasis**						0.363						0.265
Yes	25	12	48.00	13	52.00		40	12	30.00	28	70.00	
No	651	349	53.61	302	46.39		148	35	23.65	113	76.35	
**Coal-producing area**						**0.046**						0.329
Yes	403	204	50.62	199	49.38		119	28	23.53	91	76.47	
No	273	157	57.51	116	42.49		69	19	27.54	50	72.46	
**Fuyuan County origin**						**0.006**						0.396
Yes	197	90	45.69	107	54.31		57	13	22.81	44	77.19	
No	479	271	56.58	208	43.42		131	34	25.95	97	74.05	
**Xuanwei County origin**						0.179						0.318
Yes	143	71	49.65	72	50.35		47	10	21.28	37	78.72	
No	533	290	54.41	243	45.59		141	37	26.24	104	73.76	
**Total**	676	361	53.40	315	46.60		188	47	25.00	141	75.00	

Similarly, we analyzed the frequency of *EGFR* gene mutations in the adenocarcinoma subgroup. The *EGFR* gene mutation rate in adenocarcinoma patients was found to be 49.67% (376/757). Female (*p* < 0.001), non-smokers (*p* < 0.001), patients from the central Qujing City (*p* = 0.027), non-brain metastasis (*p* = 0.023), tissue specimens (*p* < 0.001), patients from the coal-producing areas (*p* = 0.004) and non-Fuyuan county origin (*p* = 0.005) and non-Xuanwei county origin (*p* = 0.027) might be associated with a significantly higher *EGFR* gene mutation rate (Table 5).

**Table 5:** Incidence of *EGFR* gene mutation in adenocarcinoma subgroup

	Sum total	Positive		Negative		*p*
		N	%	N	%	
**Age**						0.868
< 65	605	302	49.92	303	50.08	
65–75	138	68	49.28	70	50.72	
> 75	14	6	42.86	8	57.14	
**Gender**						**0.000**
Male	343	137	39.94	206	60.06	
Female	414	239	57.73	175	42.27	
**Smoking history**						**0.000**
Yes	170	60	35.29	110	64.71	
No	587	316	53.83	271	46.17	
**Family history of malignant tumors**						0.117
Yes	149	67	44.97	82	55.03	
No	608	309	50.82	299	49.18	
**Tumor site**						0.316
Left	278	144	51.80	134	48.20	
Right	459	225	49.02	234	50.98	
Bilateral	20	7	35.00	13	65.00	
**Regional distribution**						**0.027**
Central	199	117	58.79	82	41.21	
East	227	96	42.29	131	57.71	
Northeast	180	80	44.44	100	55.56	
Southeast	9	6	66.67	3	33.33	
South	14	6	42.86	8	57.14	
West	20	10	50.00	10	50.00	
Northwest	8	5	62.50	3	37.50	
Southwest	12	5	41.67	7	58.33	
North	59	36	61.02	23	38.98	
Other	29	15	51.72	14	48.28	
**Ethnic group**						0.683
Han	753	374	49.67	379	50.33	
Yi	4	2	50.00	2	50.00	
**TNM staging**						0.278
I-IIIa	582	293	50.34	289	49.66	
IIIb-IV	175	83	47.43	92	52.57	
**Brain metastasis**						**0.023**
Yes	51	18	35.29	33	64.71	
No	706	358	50.71	348	49.29	
**Specimen type**						**0.000**
Tissue	626	344	54.95	282	45.05	
Plasma	127	31	24.41	96	75.59	
Cytology	4	1	25.00	3	75.00	
**Coal producing area**						**0.004**
Yes	462	211	45.67	251	54.33	
No	295	165	55.93	130	44.07	
**Fuyuan County origin**						**0.005**
Yes	227	96	42.29	131	57.71	
No	530	280	52.83	250	47.17	
**Xuanwei County origin**						**0.027**
Yes	166	71	42.77	95	57.23	
No	591	305	51.61	286	48.39	
**Total**	757	376	49.67	381	50.33	

### Analysis of various kinds of EGFR gene mutations

It was observed that *EGFR* gene mutation pattern in the coal-producing areas in the Eastern Yunnan subgroup was inconsistent with the non-coal-producing areas. It was also found not to be consistent with the overall situation in Yunnan Province and even differed from the whole of China. It was worth noting that the frequency of *EGFR* gene G719X single-mutation and G719X + S768I compound double mutation in the lung cancer patients in the coal-producing areas was relatively high. In contrast, the mutation rate of L858R and 19-del were relatively low compared with various other regions (Table 6).

**Table 6:** Types of *EGFR* gene mutations identified in this study

	Coal-producing areas							Specimen type					Gender					Smoking history				
	Total		Yes		No			Tissue		Plasma and pleural effusion cells			Male		Female			Yes		No		
	N	%	N	%	N	%	*p*	N	%	N	%	p	N	%	N	%	*p*	N	%	N	%	*p*
**Sensitizing mutations**																						
L858R	135	33.09	63	27.16	72	40.91	**0.003**	119	32.96	16	34.04	0.882	45	30.00	90	34.88	0.31	14	21.88	121	35.17	**0.038**
19-del	87	21.32	41	17.67	46	26.14	**0.039**	77	21.33	10	21.28	0.993	30	20.00	57	22.09	0.62	13	20.31	74	21.51	0.830
G719X	29	7.11	23	9.91	6	3.41	**0.011**	25	6.93	4	8.51	0.923	9	6.00	20	7.75	0.51	6	9.38	23	6.69	0.614
G719X + L861X	10	2.45	4	1.72	6	3.41	0.443	10	2.77	0	0.00	0.513	3	2.00	7	2.71	0.91	1	1.56	9	2.62	0.952
L861Q	6	1.47	2	0.86	4	2.27	0.224	4	1.11	2	4.26	0.144	1	0.67	5	1.94	0.29	1	1.56	5	1.45	0.643
L858R + L833F	4	0.98	4	1.72	0	0.00	0.103	4	1.11	0	0.00	0.612	1	0.67	3	1.16	0.53	1	1.56	3	0.87	0.496
G719X + E709X	4	0.98	3	1.29	1	0.57	0.421	4	1.11	0	0.00	0.612	1	0.67	3	1.16	0.53	0	0.00	4	1.16	0.504
L861X + L833F	4	0.98	3	1.29	1	0.57	0.421	4	1.11	0	0.00	0.612	2	1.33	2	0.78	0.47	1	1.56	3	0.87	0.496
L858R + *EGFR *gene ampilification	3	0.74	2	0.86	1	0.57	0.603	3	0.83	0	0.00	0.692	1	0.67	2	0.78	0.69	0	0.00	3	0.87	0.599
* EGFR* gene ampilification	1	0.25	1	0.43	0	0.00	0.569	1	0.28	0	0.00	0.885	1	0.67	0	0.00	0.37	1	1.56	0	0.00	0.157
G719X + L858R	1	0.25	1	0.43	0	0.00	0.569	1	0.28	0	0.00	0.885	0	0.00	1	0.39	0.63	0	0.00	1	0.29	0.843
L833V + H835L	1	0.25	1	0.43	0	0.00	0.569	1	0.28	0	0.00	0.885	1	0.67	0	0.00	0.37	1	1.56	0	0.00	0.157
18-del	1	0.25	0	0.00	1	0.57	0.431	1	0.28	0	0.00	0.885	1	0.67	0	0.00	0.37	0	0.00	1	0.29	0.843
19-del + *EGFR* gene ampilification	3	0.74	0	0.00	3	1.70	0.079	3	0.83	0	0.00	0.692	1	0.67	2	0.78	0.69	1	1.56	2	0.58	0.401
L858R + E709X	1	0.25	0	0.00	1	0.57	0.431	1	0.28	0	0.00	0.885	0	0.00	1	0.39	0.63	0	0.00	1	0.29	0.843
L861Q + L838V	1	0.25	0	0.00	1	0.57	0.431	1	0.28	0	0.00	0.885	1	0.67	0	0.00	0.37	1	1.56	0	0.00	0.157
**Resistance mutations**																						
20INS	14	3.43	5	2.16	9	5.11	0.104	13	3.60	1	2.13	0.923	6	4.00	8	3.10	0.63	3	4.69	11	3.20	0.820
S768I	5	1.23	5	2.16	0	0.00	0.058	4	1.11	1	2.13	0.459	2	1.33	3	1.16	0.61	1	1.56	4	1.16	0.576
T790M	3	0.74	3	1.29	0	0.00	0.183	1	0.28	2	4.26	**0.036**	2	1.33	1	0.39	0.31	1	1.56	2	0.58	0.401
L747S	1	0.25	0	0.00	1	0.57	0.431	1	0.28	0	0.00	0.885	0	0.00	1	0.39	0.63	0	0.00	1	0.29	0.843
**Combination of sensitizing and resistance mutations**																						
G719X + S768I	68	16.67	56	24.14	12	6.82	**0.000**	61	16.90	7	14.89	0.729	30	20.00	38	14.73	0.17	14	21.88	54	15.70	0.223
S768I + L858R	4	0.98	3	1.29	1	0.57	0.421	4	1.11	0	0.00	0.612	2	1.33	2	0.78	0.47	1	1.56	3	0.87	0.496
19-del + T790M	3	0.74	1	0.43	2	1.14	0.397	2	0.55	1	2.13	0.308	2	1.33	1	0.39	0.31	1	1.56	2	0.58	0.401
L858R + T790M	3	0.74	1	0.43	2	1.14	0.397	3	0.83	0	0.00	0.692	0	0.00	3	1.16	0.25	0	0.00	3	0.87	0.599
19-del + S768I	1	0.25	1	0.43	0	0.00	0.569	1	0.28	0	0.00	0.885	0	0.00	1	0.39	0.63	0	0.00	1	0.29	0.843
G719X + S768I + L858R	1	0.25	1	0.43	0	0.00	0.569	1	0.28	0	0.00	0.885	1	0.67	0	0.00	0.37	1	1.56	0	0.00	0.157
G719X + S768I + *EGFR* gene ampilification	1	0.25	1	0.43	0	0.00	0.569	1	0.28	0	0.00	0.885	0	0.00	1	0.39	0.63	0	0.00	1	0.29	0.843
L858R + 20INS	1	0.25	1	0.43	0	0.00	0.569	1	0.28	0	0.00	0.885	1	0.67	0	0.00	0.37	0	0.00	1	0.29	0.843
G719X + D761Y + *EGFR* gene ampilification	1	0.25	1	0.43	0	0.00	0.569	1	0.28	0	0.00	0.885	1	0.67	0	0.00	0.37	0	0.00	1	0.29	0.843
19-del + T790M + C797S	1	0.25	0	0.00	1	0.57	0.431	0	0.00	1	2.13	0.115	0	0.00	1	0.39	0.63	0	0.00	1	0.29	0.843
G719X + S768I + T790M	1	0.25	0	0.00	1	0.57	0.431	0	0.00	1	2.13	0.115	0	0.00	1	0.39	0.63	0	0.00	1	0.29	0.843
L858R + T790M + *EGFR* gene ampilification	1	0.25	0	0.00	1	0.57	0.431	0	0.00	1	2.13	0.115	1	0.67	0	0.00	0.37	0	0.00	1	0.29	0.843
**No EGFR targeted therapies mutations**																						
V769fs,S1042I,H773L,V774M,G983W,D1127fs,R973Q,I926L,A1118V	8	1.96	5	2.16	3	1.70	0.521	8	2.22	0	0.00	0.372	4	2.67	4	1.55	0.68	1	1.56	7	2.03	0.802

**Fig. 2:**
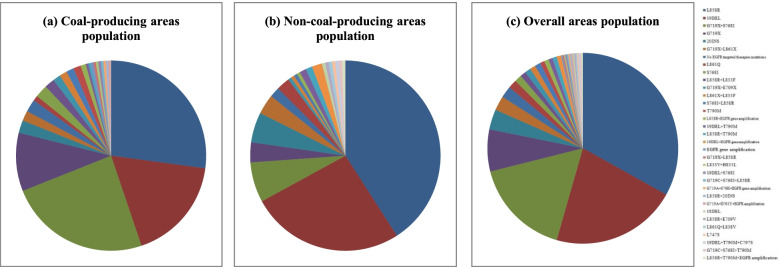
*EGFR* gene mutation spectrum in **a** coal-producing areas, **b** non-coal-producing areas, **c** overall area population. The lung cancer patients in Yunnan eastern coal-producing belts displayed more unusual G719X, G719X + S768I, but relatively lesser L858R and 19-del mutations compared to the non-coal-producing belts patients

In this study, among the 408 lung cancer patients with *EGFR* gene mutations in Eastern Yunnan, 87 types of *EGFR* gene mutation were observed. There were 61 distinct types of *EGFR* gene mutation in 232 lung cancer patients from the coal-producing areas and 43 different kinds of *EGFR* gene mutation in 176 patients of the non-coal-producing areas. The results revealed that the distribution of *EGFR* gene mutation types in the lung cancer patients of the coal-producing areas and the non-coal-producing was statistically significantly different (*p* = 0.005), whereas the diversity of *EGFR* gene mutation types in the coal-producing areas lung cancer patients was significantly more than that of the non-coal-producing lung cancer patients (Fig. 2).

In total, we had detected *EGFR* gene mutations in 408 patients. The most prevalent mutations were L858R point mutation and 19-del, with 135 cases (33.09%) and 87 cases (21.32%), respectively. In addition, 238 samples (69.12%) showed single mutations, and 126 samples (30.88%) displayed compound mutations. Of the 408 patients, 291 cases (71.32%) exhibited sensitizing mutations for the molecular-targeted drugs, 23 cases (5.64%) displayed resistance mutations in the conventional sense, 86 patients (21.08%) showed both resistance and sensitizing mutations, whereas there were no available molecular-targeted drugs for the remaining eight cases with *EGFR* gene mutation (1.96%), (Table 6). Moreover, 12 patients were found to carry an *EGFR* T790M mutation, of which 3 cases were that of T790M single mutation, 3 cases were 19-del + T790M compound mutation, 3 cases were L858R + T790M compound mutation, 1 case was that of a 19-del + T790M + C797S compound mutation, and 1 case was a G719X + S768I + T790M compound mutation, and 1 case was a L858R + T790M + *EGFR* gene amplification compound mutation. Overall, 165 patients had received EGFR-TKIs treatment, and 50 patients who had undergone EGFR-TKIs therapy belonged to wild-type *EGFR*. EGFR-TKIs administered to these patients were Gefitinib Tablets (250 mg once a day), Icotinib Hydrochloride Tablets (125 mg 3 times a day), Erlotinib Hydrochloride Tablets (150 mg per day, 2 times per day), Anlotinib Hydrochloride Capsules (12 mg once a day), Afatinib dimaleate Capsule (40 mg once a day), Osimertinib (80 mg once a day), and Almonertinib Mesilate Tablets (110 mg once a day). Apart from them, the remaining 699 patients had never experienced EGFR-TKIs treatment previously.

We also analyzed whether the type of specimen, patients from coal-producing areas, gender, and smoking history could potentially affect *EGFR* gene mutation type distribution. Our analysis demonstrated that the distribution of *EGFR* gene mutation type in the coal-producing regions of east Yunnan was significantly different from that in other regions of Yunnan province. First of all, the most critical distinction was that the rate of *EGFR* gene G719X (*p* = 0.011) single mutation and G719X + S768I (*p* < 0.001) double compound mutation in the lung cancer patients of Yunnan eastern coal-producing regions was meaningfully more unusual than that of patients in the non-coal-producing regions, where no coal is produced and there is no substantial pollution as a result of from the coal mining. Secondly, the drug-sensitive mutations such as L858R + L833F, G719X + E709X, L861X + L833F, L858R + *EGFR* gene amplification, *EGFR* gene amplification, G719X + L858R, and L833V + H835L were found to be significantly more than those in the non-coal-producing areas. Third, S768I and T790M resistance mutations were likewise more commonly observed in the coal-producing areas patients. Besides, S768I + L858R, 19-del + S768I, G719X + S768I + L858R, G719X + S768I + *EGFR* gene amplification, L858R + 20INS, G719X + D761Y + *EGFR* gene amplification, and other drug-resistant and sensitive compound mutations were comparatively found to be more commonly detected. Moreover, no additional EGFR targeted therapies for certain *EGFR* gene mutations, which also maintained a relatively high mutation rate among the lung cancer patients in the coal-producing zones was observed (Table 6). In addition, lung cancer patients in the coal-producing areas, however, displayed lower mutation frequencies for L858R (*p* = 0.003) and 19-del (*p* = 0.039) compared with the non-coal-producing areas (Table 6). Based on the type of specimen analyzed, we discovered that the frequency of T790M mutation in the plasma samples was significantly higher than that in tissues (*p* = 0.036) (Table 6). There was no statistically significant variation observed in the distribution of *EGFR* gene mutation types in the distinct genders (Table 6). According to smoking history, the mutation frequency of L858R, notwithstanding, was substantially found to be more unusual than that of smokers (*p* = 0.038) (Table 6).

**Fig. 3:**
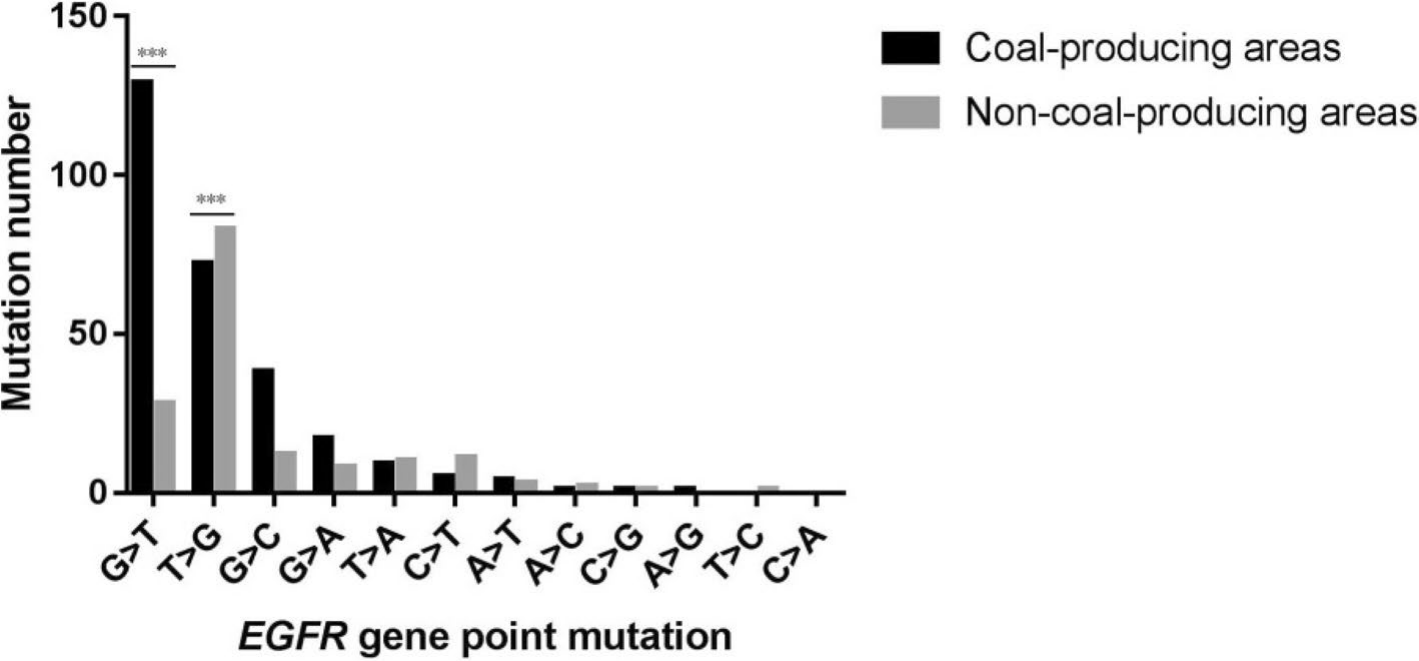
Distribution of *EGFR* gene point mutation types in the 864 patients affected with lung cancer. The difference was considered as statistically significant when *p* < 0.05 (bar chart marked with *). *P*-values were set at 0.01 (*p* < 0.01) for the highly significant differences (bar chart marked with **), and 0.001 (*p* < 0.001) for extremely significant difference (bar chart marked with ***)

A total of 574 *EGFR* gene mutations were identified in all 864 samples which were analyzed, and the dominant mutation type was base substitution (point mutation) (75.95%) (Supplementary Table 1). The frequency of transversion mutations which included mutation of guanine (G) to thymine (T) (G > T) was 157 times, and T > G was 155 times in the *EGFR* gene (*p* < 0.001) (Fig. 3). In this study, *TP53* gene exons were sequenced in 472 lung cancer patients, and we detected *TP53* mutations in 185 patients (39.19%). Among 279 lung cancer patients from the coal-producing areas, 120 patients harbored *TP53* gene mutation (43.01%), and sixty-one counts G > T point mutations were detected in 120 patients (50.83%). Among 193 lung cancer patients from the non-coal-producing areas, 65 patients harbored *TP53* gene mutations (33.68%), and nineteen counts G > T point mutations were detected in 65 patients (29.23%) (data not shown). Chi-square test analysis showed that the frequency of G > T point mutation of the *TP53* gene in lung cancer patients in the coal-producing areas was significantly higher than in the non-coal-producing areas (*p* = 0.005). Our results further confirmed that the G > T point mutation of the *TP53* gene in lung cancer patients was related to coal production.

## Discussion

In the present study, we have analyzed the potential relationship between *EGFR* gene mutations and clinical characteristics in patients with lung cancer in the coal-producing areas of East Yunnan Province.

### Study on the frequency of EGFR gene mutation in the coal-producing areas of East Yunnan

Multi-center research indicated that the overall mutation rate of the *EGFR* gene in the Asia–Pacific region lung cancer patients was approximately 39.6%. The mutation rate of *EGFR* gene in the lung cancer patients in each country was 38.1% (mainland China), 48.2% (Hong Kong, China), 53.3% (Taiwan, China), 28.7% (Indonesia), 30.2% (Japan), 35.8% (South Korea), 45.7% (Malaysia), 38.9% (Philippines), 42.9% (Singapore), 45.1% (Thailand), and 36.0% (Vietnam), respectively [14]. In this study, the total mutation rate of the *EGFR* gene in the lung cancer patients of East Yunnan Province was noted to be 47.22%, the highest mutation rate in the tissue samples was 53.40%, and the mutation rate in adenocarcinoma patients was 49.67%. The *EGFR* gene mutation rate in the tissue specimens and adenocarcinoma patients was comparatively higher, corresponding to the relevant research reports in other parts of Asia [10, 15, 16]. It was found that large population of the mutant tumor cells were present in the tissue specimens and *EGFR* mutation was primarily specific for lung adenocarcinoma. Furthermore, we discovered that the lung cancer patients’ *EGFR* gene mutation frequency in the coal-producing areas was markedly lower than that in the non-coal-producing areas, which may be associated with the differences in the primary driving genes responsible for the occurrence and development of the lung cancer in varying regions, and may be possibly related to the unique pathogenesis of the lung cancer caused by environmental pollution in Yunnan eastern coal-producing areas. Similar phenomena have also been reported in Xuanwei county of East Yunnan province [12, 13]. However, further research is required to analyze the relationship between *EGFR* gene mutations in the coal-producing areas and the mechanisms of lung cancer. Additionally, another study showed that the *EGFR* gene mutation frequency of patients with the different stages of lung cancer was markedly different. The mutation frequency of the patients with advanced and brain metastases was found to be significantly higher than that of patients with early cancer and non-brain metastasis [17, 18]. In this study, *EGFR* gene mutation frequency of the patients with non-brain metastasis was 50.88%, whereas that of the patients with brain metastasis were merely 33.33% (Table 1). The multivariate regression analysis further confirmed that the non-coal-producing areas, non-Fuyuan county origin, non-Xuanwei city origin, and stage Ia affected *EGFR* gene mutations (Table 2), which was consistent with domestic studies [12]. In our research, early lung cancer patients displayed a higher *EGFR* gene mutation frequency, which may be related to the varying clinical stages of lung cancer patients in the different studies. The vast majority of the patients in this study were from thoracic surgery who were often diagnosed with early cancer but had no distant metastasis. It is straightforward to get tested the tissue specimens for *EGFR* gene mutation during the thoracic surgery. As a result, most of the cases we analyzed were belonged to early non-brain metastases lung cancer patients’ tissue specimens. However, gender, smoking history, pathological type, brain metastasis, and specimen type were not important factors affecting *EGFR* gene mutations in the multivariate regression analysis, which might be associated with the confounding factors among the various aspects or the limited number of cases. It might be still necessary to further expand the sample size or conduct multi-center clinical research to confirm our current findings.

### Study on the mutation types of EGFR gene in coal-producing areas in Eastern Yunnan

In this study, the specific genotypes of complex *EGFR* gene mutations (double or multiple concomitant *EGFR* gene mutations) was observed to be diverse, which can be single sensitizing mutation, single resistance mutation, sensitizing mutation combined with sensitizing mutation, or sensitizing mutation combined with the resistance mutation thus forming a maximum of four distinct complex mutations at the different mutation sites. We detected 81 different kinds of *EGFR* genotypes in all 864 lung cancer patients. There were 61 *EGFR* gene mutation types among 232 lung cancer patients in the coal-producing areas and 43 *EGFR* gene mutation types among 176 lung cancer patients in the non-coal-producing areas. The statistical analysis results clearly suggested that the diversity of *EGFR* gene mutation types in the coal-producing areas was significantly greater than that in the non-coal-producing areas (*p* = 0.005). This finding further confirmed that environmental pollution caused by the coal production and coal consumption may significantly increase the diversity of driver gene mutations of the lung cancer patients [15, 19].

### Common mutations 19-del and L858R

Several reports have shown noticeable differences in *EGFR* gene mutation patterns in different regions worldwide. For instance, *EGFR* gene mutation patterns in Southeast Asian countries such as Singapore (48.6%) [20], Thailand (48.3%) [21], Malaysia (23.5%) [22], Indonesia (45.95%) [23], and Vietnam (44.4%) [24] were primarily based on exon 19-del. In addition, there was no significant difference between exon 19-del (40.0%) and L858R (40.0%) in Myanmar [25]. In China, the different types of *EGFR* gene mutations remained primarily restricted to exons 19-del in Yunnan (40.0%) [13], Guizhou (52.5%) [26], Guizhou Zunyi (47.46%) [27], and Guangxi (54.6%) [28]. Still, Hong Kong (50.5%) [29], Taiwan (52.6%) [30], Yunnan Qujing (24.28%) [31], Sichuan (42.5%) [32], Hunan (62.1%) [33], as well as Hubei (15.9%) [34] and were dominated by L858R point mutations. However, the *EGFR* gene mutation pattern in Guangdong was observed to be almost the same as the rest of China (23.0% vs. 24.1%) [35]. Guangdong is a province with a large migrant population, and the influx of people from all over country might effectively dilute the variations in the frequency of *EGFR* gene mutations. The current study found that the most common *EGFR* gene mutations in the eastern region of Yunnan were single L858R point mutations (33.09%) and 19-del (21.32%) (Table 6). Interestingly, the L858R mutation frequency of the *EGFR* gene (27.16%) was significantly higher than 19-del (17.67%) in the coal-producing regions, thereby further confirming that the type of *EGFR* gene mutation in the coal-producing areas in Eastern Yunnan was significantly different from other regions in Yunnan province. The researchers are in general agreement that the main influential factors contributing to the differences in *EGFR* gene mutation of the lung cancer patients in Eastern Yunnan were at least in part generated from the human-induced environmental pollution during the process of mining [12, 31, 36–38]. A number of prior studies have described that the main factor contributing to the high lung cancer incidence of East Yunnan was that the local residents were exposed to indoor air pollution from the coal combustion and outdoor heavy metal as well as organic-matter pollution from coal mining [39–41]. The delicate particulate matter in the polluted air significantly reduced the cell survival rate of the *EGFR* mutant (19-del) human lung adenocarcinoma cell lines HCC827 [7]. However, the possible relationship between L858R mutation and coal-burning particles has not yet been reported or validated, and the underlying mechanisms require further in-depth studies.

Lung cancer patients with different *EGFR* gene mutations can experience a differential effect of the treatment. Traditionally, the patients with *EGFR* gene L858R and 19-del mutations have been classified as EGFR-TKIs (including gefitinib, erlotinib, and afatinib) sensitive group [42]. However, the two randomized phase III trials LUX-Lung 3 and LUX-Lung 6 found that afatinib exhibited survival benefits only for 19-del mutation but not for the patients with L858R mutation [43]. In addition, 19-del included at least 30 different variants. The rare mutation delE746_S752insV may be insensitive to gefitinib [44–46]. For instance, Chung et al. (2012) reported that the ORR to first-generation EGFR-TKIs in patients harboring 19-del starting at 746, 751, or 752 was lower than the deletions at 747 site [47]. In brief, different mutation sites in the common mutations also exhibited different sensitivity to the same targeted drugs. Here, we found that there were rare mutation sites present in the common mutation types of the *EGFR* gene, yet the real-time fluorescent quantitative PCR approach was not able to precisely detect the site-specific mutation of the *EGFR* gene. It is therefore necessary to use the NGS method to accurately screen the entire exome to determine rare mutation sites that can enable the patients to benefit from the precision treatment.

The common *EGFR* gene mutations detected were L858R and 19-del in patients of Qujing origin (eastern Yunnan province) were indeed relatively low. Suda et al. (2021) examined *EGFR* gene mutations in 5780 Japanese lung cancer patients, 2410 patients had *EGFR* gene mutations (41.7%), 983 patients had 19-del mutations (40.8%), whereas 1170 patients displayed L858R point mutation (48.5%) [48]. The overall mutation rate of the *EGFR* gene in lung cancer patients of Qujing origin was 47.22%, 19-del was 21.32%, and L858R was 33.09%. The mutation rate of L858R and 19-del in Qujing lung cancer patients was markedly lower than in Japanese patients. Moreover, we found that the mutation frequency of 19-del (26.14% vs. 17.67%, *p* = 0.039) and L858R (40.91% vs. 27.16%, *p* = 0.003) in lung cancer patients in the non-coal-producing areas of Qujing was significantly higher than that in patients in coal-producing areas (Table 6), but both were substantially lower than that in Japanese lung cancer patients.

### *Uncommon mutations G719X and G719X* + *S768I were not rare in the coal-producing areas in Eastern Yunnan*

The most striking result to emerge from the analysis was that G719X (9.91%, *p* = 0.011) and G719X + S768I (24.14%, *p* = 0.000) mutation frequencies in the lung cancer patients of the coal-producing regions of East Yunnan were significantly higher than that in the non-coal-producing region patients that other related studies have previously reported [12, 31, 36–38]. For instance, multi-center study showed that G719X single mutation and G719X + S768I compound double mutation respectively accounted for approximately only 1.94% and 0.59% of all *EGFR* gene mutations in the Chinese population [49]. Nevertheless, the G719X + S768I (24.14%) compound double mutation frequency was relatively close to the primary common mutation L858R (27.16%) but higher than 19-del (17.67%) in the lung cancer patients of the coal-producing area of East Yunnan (Table 6). In addition, previous studies have demonstrated that the lungs exposed to coal-burning particles were a significant risk factor affecting *EGFR* gene mutations in the lung cancer patients. The researchers hypothesized that *EGFR* exon 18 and 21 mutations were more sensitive to coal combustion emissions [7]. Furthermore, we found that the exon 20 mutation was also sensitive to the coal combustion emissions as a potential supplement to the molecular mechanisms of high incidences of lung cancer in the coal-producing area of East Yunnan. However, very little information was found in the literature on the various environmental factors related to the high incidence of lung cancer in the coal-producing areas and hence further research in this area is urgently needed. The burning of the coal led to the formation of unique molecular markers based on rare mutations of G719X and G719X + S768I in the coal-producing area of East Yunnan. The lung cancer patients with different *EGFR* gene mutations can display different sensitivities to EGFR-TKIs. We also speculate that the coal combustion particles may also be related to the EGFR-TKIs response.

A number of in vivo and in vitro experiments have confirmed that the first-generation (Gefitinib and Erlotinib), the second-generation (Afatinib, Dacomitinib, Lenatinib), and the third-generation EGFR-TKIs (Osimertinib and Rociletinib) can display therapeutic effects on patients with G719X and G719X + S768I mutations. Among them, the lung cancer patients with G719X + S768I compound double mutation were observed to have an ORR of only 53% when treated with gefitinib. However, the lung cancer patients were reported to be administered afatinib for G719X, S768I, and G719X + S768I mutations with good therapeutic effect, which increased ORR up to 77.1%-100% the maximal inhibitory concentration only the highest was 0.9 nM [42, 50]. Li et. al. (2017) reviewed the carcinogenic and drug susceptibility mechanisms caused by the G719X mutation based on the findings of protein structure, functions, cell viability, and animal experiments. The results showed that the G719X mutation was only moderately sensitive to TKIs, with an average response rate of 35.1% [51]. Moreover, a study by D’ Souza (2020) showed that the median survival period (6 months vs. 38 months) and PFS period (8 months vs. 44 months) of the patients with S768I mutation were markedly shorter than that of patients with other *EGFR* gene mutations, which indicated that the patients with S768I mutation caused substantial progressive disease and poor prognosis [52]. In additional, clinical studies have shown that erlotinib and gefitinib might have differential effects on the lung cancer patients with G719X/S768I single mutation and compound mutation. In the lung cancer patients with G719X + S768I compound mutation subgroup, the ORR and PFS were found to be 68.4% and 11.9 months, respectively, which was significantly superior to that in the G719X single mutation subgroup (36.8% and 6.3 months), even close to those with 19-del mutation (65.3% and 13.5 months) [53]. Moreover, Kutsuzawa (2020) study showed that lung adenocarcinoma patients harboring both G719X and S768I mutations of the *EGFR* gene can be successfully treated with afatinib and had PFS for 17 long months [54]. It was postulated that G719X + S768I compound double mutations may cause a favorable change in the three-dimensional structure of the EGFR protein, which can effectively enhance the binding force between afatinib and EGFR. However, it is necessary to analyze further that how the G719X/S768I single mutation and compound mutations can change the crystal structure and function of EGFR protein. In addition, afatinib was also particularly effective for the patients with 18-del, E709K, L861Q, or exon 19 insertion mutations [42]. In the coal-producing areas of East Yunnan, the G719X single mutation and G719X + S768I compound double mutations were principal *EGFR* gene types identified in the lung cancer patients, but other sensitive mutations (18-del, L861X, L833F, E709X, and *EGFR* gene amplification) were diverse. The lung cancer patients would be thus expected to benefit more from afatinib treatment than others TKIs. Our study provides additional support for application of afatinib as first-line targeted therapy for the lung cancer with G719X, S768I, and L861Q mutations in the latest National Comprehensive Cancer Network (NCCN) guidelines (2022.v1 edition).

### Common resistance mutation T790M

It is well-established that *EGFR* gene T790M is the most common mutation associated with acquired resistance to EGFR-TKIs. In this study, single T790M mutation frequency was 0.74% (3/864), and compound T790M mutation frequencies were 2.21% (9/864), which were almost similar to the findings of other East Asian countries. After collecting enough T790M mutation cases, we aim to further analyze whether there might be significant differences between T790M in the coal-producing areas and the non-coal-producing areas. Of the 601 NSCLC patients with *EGFR* gene mutations in South Korea, 13 patients (2.2%) displayed T790M single or compound mutations, and four patients exhibited T790M single mutation [55]. Among the 12 patients with T790M mutation in this study, six received EGFR-TKIs treatment, one received almonertinib mesylate tablets, and the rest of the other received osimertinib after drug resistance. The remaining four refused to take any targeted therapy.

### *EGFR gene G* > *T point mutation*

DeMarini et al. (2002) found that benzopyrene, a carcinogen produced by coal-burning, could lead to a G > T transversion mutation of the *TP53* gene in the lung cancer patients [56]. Excessive G > T transversion mutations in the *TP53* gene have been identified as the “molecular signature” of the various tobacco smoke mutagens in smoking-related lung cancer [57]. This study found that excessive G > T and T > G transversion mutations in *EGFR* gene were unique molecular mutation characteristics in lung cancer patients in the coal-producing areas, and the reasons may be as follows: (1) polycyclic aromatic hydrocarbons (PAHs) are the main carcinogens found in the emissions from coal-burning, which can interact with DNA to form polycyclic aromatic hydrocarbon dihydrodiol epoxide (PAH-DNA adducts). These adducts can combine with the nucleophilic group of the exocyclic amino group in the guanine (G), which then pairs with thymine (T) instead of cytosine (C) during the DNA replication process. Transversion mutations G > T were mainly induced in *EGFR* gene mutations [58]. (2) PAH-DNA adducts were present in the human tissue DNA, which was exposed to the tobacco smoke [59]. (3) In addition, compared with non-coal-producing lung cancer patients, G > T transversion mutation frequency was found to be significantly increased in the coal-producing lung cancer [38].

### Lung cancer in coal-producing area and the change of local people's lifestyle

As early as the 1970s, researchers found that indoor coal-burning pollution might be responsible for the high incidence of lung cancer in the coal-producing areas of Eastern Yunnan province of China. There are abundant coal reserves in eastern Yunnan, and the local rural residents have been regularly burning coal from a long time for heating and cooking at home. However, there are no air intakes or chimneys in the fire ponds that can burn coal, and thereby the soot generated by coal accumulates indoors, causing indoor air pollution and contributing to the highest incidence of lung cancer in rural areas globally [60]. Since the 1980s, the coal-producing regions in eastern Yunnan have carried out large-scale projects to adopt the use of ovens and stoves, which has led to a significant decrease in the concentration of indoor particulate matter and carcinogenic PAHs. However, in both the males and females, the death rate from the lung cancer has not decreased as expected in recent years [6]. We followed up with the 864 lung cancer patients in this study by telephone. We found that 310 of them had now switched to electricity, 121 people now use mixed electricity and smokeless coal, 285 people use mixed electricity and smoky coal, 83 people use mixed electricity and wood, nine people use smokeless coal, 43 people use smoky coal and only one person uses wood for cooking and heating (Supplementary Table 1) still have lung cancer. The possible causes are: (1) In this study, patients over 40 years old in the rural areas in the coal-producing areas still mainly use coal in winter, although they primarily use wood as well as electricity for the cooking in spring, summer, and autumn. In addition, these patients still use smoky coal before they are 20 years old and have been exposed to indoor air pollution caused by coal burning for decades [41]. (2) Although many rural families have installed chimneys, the height is only slightly more than one meter. When the wind blows after the soot is discharged, it is easy to pour it back inwards. As far as the whole area of eastern Yunnan is concerned, the risk of the lung cancer is still dominated by indoor pollution [61]. (3) Most of the coal-producing areas in eastern Yunnan are surrounded by the mountains. During the mining of the local coal mines, water and air are polluted, and the polluted air accumulates over the village for a long period of time, which is not conducive to facilitate the diffusion of soot [62].

### Limitations

There are two major limitations associated with our study. The first was the lack of follow-up. We collected data on *EGFR* gene mutations and clinical characteristics of 864 patients with the lung cancer. 522 cases were from the coal-producing areas, and 342 were from the non-coal-producing areas in Eastern Yunnan. It was noted that the types of *EGFR* gene mutation in the lung cancer patients of coal-producing areas were significantly different in other patients in Yunnan Province. However, the research related to the efficacy of EGFR-TKIs on the lung cancer patients of the coal-producing regions has not yet been reported. To further provide evidence and reference for individualized treatment and pathogenesis in local lung cancer cases, we will continue to follow-up and look forward to analyze the various possible reasons for the high incidence of lung cancer and the efficacy of EGFR-TKIs treatment in the coal-producing areas of East Yunnan. Secondly, *EGFR* gene activating mutation could effectively alter the configuration of kinase to increase the receptor activity and influence the efficacy of TKI. Suzuki et al. (2008) found that the length of the first intron CA repeat polymorphism of the *EGFR* gene was inversely related with EGFR protein expression level in the lung carcinoma [63]. It has been proved that compared with EGFR protein expression, *EGFR* gene mutation may be a relatively better predictor of TKIs therapy. However, *EGFR* gene mutation, amplification, and protein expression might not be directly linked to each other. We should thus further detect the activation of EGFR protein in both the wild-type and different mutation types. It may help us to understand the possible mechanisms of lung cancer development in the coal-producing areas of East Yunnan.

## Conclusions

This study has enormous significance in establishing the potential correlation between routine using NGS for *EGFR* gene mutation diagnosis and clinical practice in the lung cancer patients. The *EGFR* gene mutation profile of the lung cancer patients of coal-producing areas in Eastern Yunnan was found to be remarkably different from that of non-coal-producing regions. The frequencies of G719X and G719X + S768I mutations were significantly higher than the overall Chinese population, but L858R point mutation and exon 19 deletion mutation frequencies was markedly lower than the overall Chinese population. Moreover, our results have also strengthened the evidence for the effectiveness of the afatinib (second-generation EGFR inhibitor) as first-line treatment option in the population of the coal-producing areas in Eastern Yunnan of Southwestern China.

## Supplementary Information

**Additional file 1:** **Supplementary Table 1.** Clinical demography, pathology, regions distrbution and EGFR gene mutation details of all participants.

## Data Availability

The dataset used and analyzed during the current study are available from the corresponding author, on reasonable request.

## Abbreviations

EGFR: Epidermal growth factor receptor
NSCLC: Non-small-cell lung cancer
LUAD: Lung adenocarcinoma
PAHs: Polycyclic aromatic hydrocarbons
NGS: Next-generation sequencing
EGFR-TKI: EGFR tyrosine kinase inhibitor
ORR: Objective response rate
DCR: Disease control rate
PFS: Progression-free survival
